# Hypopituitarism and pregnancy: clinical characteristics, management and pregnancy outcome

**DOI:** 10.1007/s11102-021-01196-7

**Published:** 2021-11-30

**Authors:** Anna Aulinas, Nicole Stantonyonge, Apolonia García-Patterson, Juan M. Adelantado, Carmen Medina, Juan José Espinós, Esther López, Susan M. Webb, Rosa Corcoy

**Affiliations:** 1 Department of Endocrinology and Nutrition, Hospital de la Santa Creu i Sant Pau, Barcelona, Spain; 2 Centro de Investigación Biomédica en Red de Enfermedades Raras (CIBERER Unidad 747), ISCIII, Barcelona, Spain; 3 Department of Medicine, University of Vic - Central University of Catalonia, Vic, Barcelona, Spain; 4 Institut de Recerca, Hospital de la Santa Creu i Sant Pau, Barcelona, Spain; 5 Department of Medicine, Universitat Autònoma de Barcelona, Barcelona, Spain; 6 Department of Gynecology and Obstetrics, Hospital de la Santa Creu i Sant Pau, Barcelona, Spain; 7 Department of Pediatrics, Hospital de la Santa Creu i Sant Pau, Barcelona, Spain; 8 Centro de Investigación Biomédica en Red de Bioingeniería, Biomateriales y Nanomedicina (CIBERBBN), ISCIII, Madrid, Spain

**Keywords:** Hypopituitarism, Pregnancy, Hormone replacement therapy, Outcome

## Abstract

**Purpose:**

To describe the clinical characteristics, management and pregnancy outcome of women with prepregnancy hypopituitarism (HYPO) that received care at our center.

**Methods:**

Retrospective study describing 12 pregnancies in women with prepregnancy HYPO (two or more pituitary hormonal deficiencies under replacement treatment) that received care during pregnancy at Hospital Santa Creu i Sant Pau. Clinical characteristics, management and pregnancy outcome were systematically collected.

**Results:**

Average patients’ age was 35 years and HYPO duration at the beginning of pregnancy was 19 years. The most frequent cause of HYPO was surgical treatment of a sellar mass (8 pregnancies). Eight pregnancies were in primigravid women and 10 required assisted reproductive techniques. The hormonal deficits before pregnancy were as follows: GH in 12 women, TSH in 10, gonadotropin in 9, ACTH in 5 and ADH in 2. All deficits were under hormonal substitution except for GH deficit in 4 pregnancies. During pregnancy, 4 new deficits were diagnosed. The dosage of replacement treatment for TSH, ACTH and ADH deficits was increased and GH was stopped. Average gestational age at birth was 40 weeks, gestational weight gain was excessive in 9 women, 8 patients required induction/elective delivery and cesarean section was performed in 6. Average birthweight was 3227 g. No major complications were observed. Five women were breastfeeding at discharge.

**Conclusions:**

In this group of women with long-standing HYPO, with careful clinical management (including treatment of new-onset hormonal deficits) pregnancy outcome was satisfactory but with a high rate of excessive gestational weight gain and cesarean section.

## Introduction

Hypopituitarism (HYPO) defined as the loss of function of one or more pituitary hormones, is a rare condition, with a reported prevalence between 300 and 355 per million inhabitants [1, 2]. The etiology is widely diverse, of which pituitary adenoma and its treatment-derived complications are the main causes followed by trauma, vascular injury, hypophysitis and infiltrative processes, among others [3]. Clinical manifestations and decision to initiate hormonal replacement will depend on diverse factors such as sex, age, severity of damage, time of onset and the hormonal axes affected. Of note, a careful monitoring of the hormonal replacement is mandatory, since hormonal replacement interactions might occur, especially when several hormonal deficiencies exist [4]. Globally, patients with HYPO have been shown to have reduced quality of life [5] along with higher rates of infertility [6] and mortality [7].

It is well known that not only the integrity of the pulsatile secretion of gonadotropin-releasing hormone and consequently of follicle-stimulating hormone and luteinizing hormone axis is needed to achieve pregnancy, but also the interaction of all pituitary hormones [6, 8, 9]. In this context, previous studies have reported that HYPO is often associated with lower pregnancy and live birth rates compared to women with either no hormonal deficits or with isolated hypogonadotropic hypogonadism [10].

During the last decades, the remarkable advance in assisted reproductive techniques (ART) has made it possible to significantly improve the fertility rate in patients with hypopituitarism. Despite the fact that the available data on this condition is scarce, a recent review based on retrospective studies [6] reported fertility rates of 81% in women with isolated hypogonadotropic hypogonadism and between 47 and 76% in women with HYPO.

Along with the development of ART, treatment with growth hormone (GH) may have concurred to the significant improvement of fertility and pregnancy outcomes during the last years [11]. A large body of evidence supports that GH plays a fundamental role in the reproductive system [10, 12, 13]. However, the continuation of GH therapy during pregnancy remains a matter of discussion, and its use during pregnancy is not approved [11, 14]. It is well established that GH is secreted from placenta whose levels increase throughout pregnancy and progressively replace pituitary GH in the stimulation of insulin-like growth factor 1 (IGF1) [15]. Of note, placental GH levels are similar in pregnant women with GH deficiency than in those without [16].

Pregnancy in hypopituitary women has classically been considered a high-risk condition based on the association with obstetrical complications such as higher rates of miscarriage, fetal growth restriction, cesarean section and post-partum hemorrhage [17, 18]. By contrast, recent studies have shown better results; such as those reported by Correa et al. [14], who described a 100% successful pregnancy rate in a case series of five women with childhood onset hypopituitarism after an optimized hormonal replacement, with no severe obstetrical complications.

Once pregnancy is achieved, a strict follow-up is needed for dosage replacement adjustment and early diagnosis of eventual new onset hormonal deficits. However, due to the lack of data based on randomized clinical trials, the actual clinical practice recommendations [4] are based on expert opinions and on the knowledge of hormonal physiology during pregnancy.

In order to contribute to the extension of the available data, we describe the clinical aspects as well as management and pregnancy outcomes of a case series of women with HYPO followed at our center.

## Methods

This is a retrospective descriptive study of patients with HYPO that were attended at the outpatient clinic for Endocrine Disorders and Pregnancy at the Endocrinology Department in Hospital Santa Creu i Sant Pau, between October 2001 and November 2019. The study was approved by the Hospital Ethics Committee that waived the requirement of an informed consent.

### Inclusion criteria

(1) Prepregnacy HYPO defined as the deficit of at least two pituitary hormones requiring substitutive treatment and (2) confirmed pregnancy.

Clinical data were obtained from electronic medical records including visits at primary care and endocrinology and obstetrics departments. Information on the following variables was collected:

*Pituitary disease and treatment: primary diagnosis and date, treatment received. *Hormonal status before pregnancy: type and number of hormonal deficits, need of replacement therapy and dosage, plasmatic and/or urine hormone levels. *Fertility: treatment used to achieve pregnancy, if needed. *Clinical assessment during pregnancy: anthropometrics, periodic hormonal status evaluation, substitutive treatment and dosage and presence of any complication. *Delivery: gestational age, induction, oxytocin use, type of delivery, pregnancy-induced hypertension, perinatal complications. *Postpartum: complications. *Neonatal: sex, birth weight, Apgar score at 5 and 10 min, arterial and venous pH, presence of any type of breastfeeding [19] at discharge and neonatal complications.

Customized birthweight centiles were calculated using gestational related optimal weight (GROW) software [20].

Data are expressed as median (percentile 25–75) using the SPSS version 20 statistical package.

## Results

### Before pregnancy

Here we present the data of 12 pregnancies in 10 women with HYPO. The characteristics of each patient are summarized in Table 1. The most frequent cause of HYPO was a sellar mass that had undergone surgical treatment.

**Table 1:** General characteristics at beginning of pregnancy of all patients

Patient	Age (years)	Initial diagnosis	Treatment received for primary condition	Duration of HP (years)	BMI (kg/m^2^)	Weight gain during pregnancy (kg) (adequate/excessive)	Hormonal deficits & disorders	Fertility treatment
01	35	Cushing’s disease	Medical + surgery	5	17.5	19.3 (excessive)	GH, gonadotropins, TSH, ACTH, ADH*	Ovarian stimulation
02	36	Granular cell tumor	Surgery	1	23.4	12.0 (excessive)	GH, gonadotropins, TSH, ACTH, ADH	Ovarian stimulation
03	34	Craniopharyngioma	Surgery	21	29.5	15.3 (excessive)	GH, gonadotropins, TSH	None^‡^
04	35	Empty sella	None	21	26.2	8.0 (adequate)	GH, gonadotropins, TSH, ACTH	In vitro fertilization
05^#^	36	Macroprolactinoma	Medical + surgery	19	28.0	15.0 (excessive)	GH, TSH, PCOS	Clomiphene citrate
06^#^	42	Macroprolactinoma	Medical + surgery	25	30.8	19.0 (excessive)	GH, TSH. PCOS	Ovarian stimulation + intrauterine insemination
07 &	29	Pituitary agenesis	Medical	19	20.7	27.0 (excessive)	GH, gonadotropins	Ovarian stimulation
08	30	Pituitary agenesis	Medical	27	21.6	15.0(excessive)	GH, gonadotropins, TSH	Ovarian stimulation
09 &	34	Pituitary agenesis	Medical	23	22.1	19.0 (excessive)	GH, gonadotropins, TSH*	Ovarian stimulation
10	36	Craniopharyngioma	Surgery	8	29.0	17.0 (excessive)	GH, gonadotropins, TSH, ACTH, ADH*	In vitro fertilization
11	35	Non-secretory pituitary adenoma	Surgery	9	23.6	8.5 (adequate)	GH, gonadotropins, TSH, ADH, ACTH	In vitro fertilization
12	42	Cushing’s disease	Medical + surgery + radiotherapy	12	20.0	11.8 (adequate)	GH, TSH, ACTH partial*	None
All patients Median (p25–75) or (n)	35.0 (34.0–36.0)	8 masses 4 agenesis/ hypoplasia	7 Medical 8 Surgery 1 radiotherapy	19.0 (7.50–22.0)	23.5 (20.9–28.7)	15.15 (10.9–18.8)	12 GH/ 10 gonadotropins/ 2 PCOS/ 10 TSH/ 5 ACTH/ 2 ADH	6 Ovarian stimulation (1 + intrauterine insemination) 3 In vitro fertilization 1 Clomiphene citrate

*De novo deficit intrapregnancy; ^#^same patient; & same patient ^‡^patient with unexpected spontaneous ovulation, description in the first section of results

From the women with GH deficiency, 66.6% were under GH substitution therapy, with a median daily dosage of 0.51 mg (0.40–0.60). All women stopped GH treatment after pregnancy confirmation. All the other TSH, ACTH and ADH deficiencies were under substitution therapy with levothyroxine, hydrocortisone and desmopressin, respectively. No patients had concomitant hormonal hypersecretion.

Women were primigravida in 8 of the 12 pregnancies and patients with prior pregnancies had had 3 abortions and 2 live children. In order to achieve pregnancy, only 2 women did not require ART. Patient #3 was diagnosed of craniopharyngioma at 15 at another center and had postoperative deficiency of GH, TSH and gonadotropins. She received treatment with levothyroxine, had puberty induction at 17 and used long-term replacement therapy with oral estrogens and gestagens. She got spontaneously pregnant when she dropped replacement therapy for 2 months. Sixteen months after delivery and 2 months after stopping partial breastfeeding she initiated transdermal hormone therapy due to hypogonadotropic hypogonadism (estradiol 0.13 nmol/l, LH 7.9 IU/L, FSH 6.6 IU/L) with normal prolactin and severe vasomotor symptoms. Transdermal treatment was stopped 10 years after delivery, and she had regular menses for 3 years. Thus, even when this is an unusual clinical course, we consider that this patient had (intermittent) hypogonadotropic hypogonadism and had spontaneous pregnancy favored by stopping combination therapy. The situation would be parallel to that in subjects with idiopathic hypogonadotropic hypogonadism where reversal and relapse has been described, even in subjects with severe phenotype [21]. In their article, Sidhoum et al. reported that up to 22% of subjects experienced reversal during their lifetime and one of the conclusions was to emphasize the need that these patients take contraceptive precautions despite apparent infertility. Patient #6 and #7 required ART due to PCOS.

### During pregnancy

**Fig. 1:**
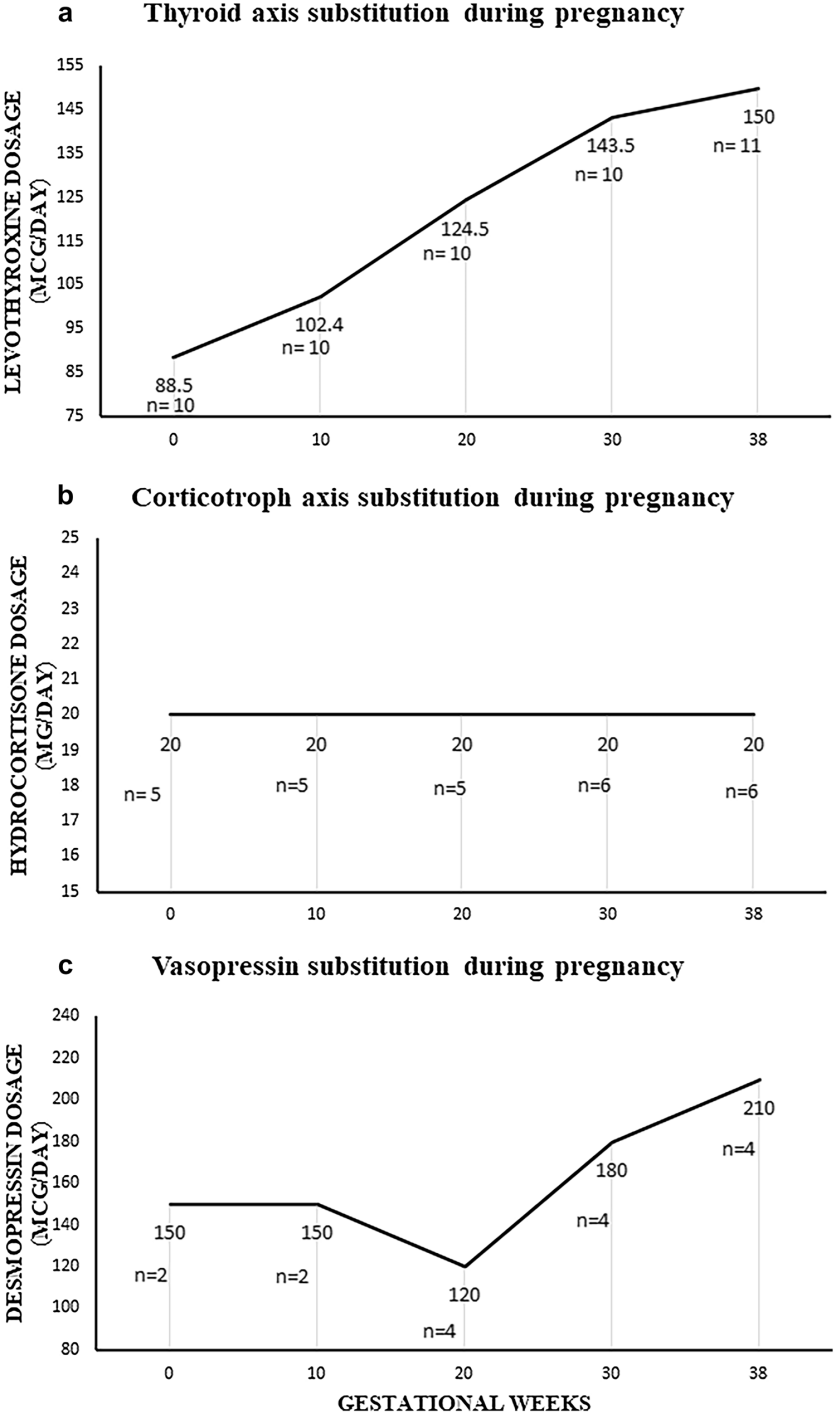
Evolution of hormonal treatment during pregnancy. Data are expressed as median dose in women receiving treatment at a specific gestational age

All but 2 patients were under follow-up at the Endocrinology Department before conception. The dosage of hormonal substitution of each hormonal axis during pregnancy is represented in Fig. 1. During pregnancy follow-up, 4 patients had to initiate treatment for a de novo deficit: one for thyroid deficit (T4 7.5 pmol/L; reference range 9–19 pmol/L) at 32 weeks, one for ACTH at 24 weeks (sinusitis in a patient with prepregnancy partial deficit) and 2 patients for ADH deficit at 18 and 21weeks (diuresis 2860 mL/24 h and 5200 mL/24 h) respectively. In those patients on levothyroxine before pregnancy, the dose during pregnancy was increased by 71.7%. Weight increase during pregnancy was excessive in 9 women according to the Institute of Medicine guidelines [22]. No patient developed apoplexy, visual impairment or any other acute complication in relation with the pituitary disease.

### Labor, delivery and pregnancy outcome

Pregnancy outcome is described in Table 2. Eight patients had labor induction/elective delivery and oxytocin was administered in about half of the patients for labor induction/augmentation. In the five patients using replacement treatment with hydrocortisone, stress dose was administered at delivery (four intravenous and one oral route). No patient developed adrenal crisis or problematic hypotension.

**Table 2:** Pregnancy, labor ad perinatal outcome of all patients

Patient	Pregnancy- induced hypertension	Gestational age at delivery (weeks)	Induction/elective delivery	Oxytocin use	Type of delivery	Newborn sex	Newborn weight (g) Birthweight centile	Apgar (1 min/ 5 min)	Cord arterial pH	Cord venous pH	Breastfeeding at discharge	Perinatal complications
01	No	37 + 4	Yes	Yes	Instrumental	Female	2495 P9	9/10	7.27	Unknown	No	No
02	No	42 + 1	Yes	Yes	Instrumental	Male	3330 P28	8/9	7.16	7.21	Yes	No
03	Yes	36 + 3	Yes	No	Cesarean section	Female	3170 P97	5/9	7.10	7.23	Yes	No
04	No	37 + 3	Yes	No	Cesarean section	Female	2990 P64	9/10	Unknown	Unknown	Unknown	No
05 #	No	42 + 2	No	Yes	Cesarean section	Female	4040 P78	9/10	Unknown	Unknown	Unknown	No
06 #	No	40 + 4	Yes	No	Cesarean section	Female	4100 P94	8/9	Unknown	Unknown	No	No
07 &	No	40 + 0	No	Yes	Instrumental	Male	3195 P31	9/10	7.15	7.24	No	No
08	No	40 + 6	No	No	Cesarean section	Female	3260 P54	9/10	7.23	7.26	Yes	No
09 &	No	37 + 3	Yes	Yes	Instrumental	Female	2540 P12	9/10	7.27	7.41	Yes	No
10	No	40 + 0	Yes	No	Cesarean section	Male	3480 P47	9/10	Unknown	Unknown	Yes	No
11	Yes	40 + 0	Yes	Yes	Eutocic	Male	3430 P60	9/10	Unknown	Unknown	No	No
12	No	40 + 5	No	Unknown	Eutocic	Male	2790 P2	9/10	7.27	7.36	No	No
All patients median (p25–75) or (n)	2 Yes 10 No	40.0 (37.4–40.8)	8 Yes 4 No	6 Yes 5 No 1 Unknown	6 Cesarean 4 Instrumental 2 Eutocic	7 Female 5 Male	3227 (2840–3467)	9.0 (8.2–9.0) 10 (9.2–10)	7.23 (7.15–7.27)	7.25 (7.22–7.37)	5 Yes 5 No 2 Unknown	12 No

Cesarean section was performed in half of the patients. Birthweight centiles of the newborns were <10 in 2 babies and >P90 also in 2 babies. No episodes of intrapartum fetal distress nor major perinatal complications were present. Five women were breastfeeding at discharge.

## Discussion

Pregnancy in women with HYPO is infrequent. It is associated with a higher risk of maternofetal complications that requires a strict follow-up by a multidisciplinary team. Due to the lack of data from randomized clinical trials, clinical guidelines [4] mainly rely on pregnancy physiology and expert recommendations. In this report, we aimed to contribute with clinical data and pregnancy outcomes in 12 pregnancies in hypopituitary women. Overall, pregnancy outcome was satisfactory, supporting the close monitoring approach prior to and during pregnancy by a specialized and multidisciplinary team.

As previously mentioned, despite the fact that new ART have notably improved fertility rates in these patients, it has been widely reported that HYPO implies a poorer pregnancy potential. In the current study, most hypopituitary women presented gonadotropin deficit and in 10 out of 12 pregnancies some type of fertility treatment was required in order to achieve conception. Unfortunately, we do not have information on non-pregnant women with HYPO, therefore we cannot provide data about the fertility rate.

GH substitution during pregnancy in women with HYPO is controversial. Some hormonal interactions may occur and close monitoring of free T4 and cortisol status is advised once GH replacement is started. GH reduces the 11β-hydroxysteroid dehydrogenase (11βHSD) type 1 activity and increases the deiodinase type 1 activity that can decrease cortisol and free T4 levels [23, 24], and upward dose adjustments of hydrocortisone and levothyroxine might be required. The contrary situation could take place during pregnancy when GH treatment is stopped. While it is known that adequate substitution prior to pregnancy is needed, because GH deficiency is related to subfertility, the current recommendation is to stop treatment once pregnancy is confirmed [4]. This recommendation is based on the physiological secretion of placental growth hormone from the 5th week of gestation [25], added to sufficient evidence regarding safety of exogenous hormone during this period. In the current study, all patients stopped GH replacement when pregnancy was confirmed. Along with several other benefits, it is generally established that despite the insulin antagonist action of GH, replacement therapy in patients with GHD, reverts almost all the metabolic alterations associated with this entity [26] by reducing fat mass, especially central adiposity and increasing lean body mass [27]. Therefore, the suspension of GH replacement at the beginning of pregnancy could have a role in the weight gain observed during follow up. It has been suggested to continue GH therapy in the first trimester and at half the dose in the second, stopping it in the last trimester when placental GH levels reach the peak [28]. Unfortunately, evidence regarding this issue ensuing from randomized controlled trials is not available.

During pregnancy, a strict follow up is essential to assess and adjust the dosage of every hormonal replacement treatment. Clinical practice guidelines on HYPO management [4] recommend to assess free T4 levels every 4–6 weeks in order to adjust levothyroxine dosage to maintain free T4 within normal ranges. Levothyroxine requirements during pregnancy might differ depending on the cause of hypothyroidism. Due to the TSH effect of hCG by binding to the TSH receptor, patients with secondary hypothyroidism could require a lower dose increment compared to those with primary hypothyroidism. Alternatively, thyroid gland may not respond appropriately to hCG due to chronic lack of TSH stimulus. In this report, the observed average increase in levothyroxine dose during pregnancy in women receiving prepregnancy treatment (71.7%) is well above reported values [29, 30], classically described as 25–30% [29]. The stop of GH treatment should have little impact considering the progressive appearance of placental GH, but if any, the effect should be in the direction of a lower increment [31]. Overall, current results do not suggest that women with hypothyroidism due to panhypopituitarism have lower requirements of levothyroxine during pregnancy.

Regarding the glucocorticoid axis adjustment, the recommendation is that during pregnancy only hydrocortisone should be used [32], since it is akin to the natural hormone, it is deactivated by the enzyme 11βHSD type 2 and cannot cross the placenta into the fetal circulation, preventing any undesirable effects of fetal exposure to glucocorticoid excess [33], contrary to dexamethasone, which is not inactivated. However, the best regime recommendation on hydrocortisone dose adjustment is still lacking. Furthermore, some hormonal replacement interactions may reduce cortisol levels, such as an increase in renal 11βHSD type 2 activity or an increased cortisol clearance due to levothyroxine therapy [4], and the previously mentioned effects of GH substitution. The suggestion from the abovementioned guideline [4] is to adjust dose after clinical judgment or to increase the hydrocortisone 20-40% in the third trimester. In the pregnancies herein reported, we observed that 3 out of the 5 pregnant women under hydrocortisone treatment did not require a dose increase during pregnancy, and the other 2 required a small increase of only 16.0% (3.2 mg/day) during the third trimester. The graph depicting median hydrocortisone dose during pregnancy (Fig. 1) is remarkably flat. It is well known that pregnancy could be considered a “physiological” state of hypercortisolism, based on the higher levels of cortisol binding globulin enhanced by placental estrogen and placental corticotropin-releasing hormone that lead to an increase of free and total plasma cortisol levels [34]. Our therapeutic decision was based on clinical judgment as the measurement of plasma cortisol levels during pregnancy is not performed as part of our regular clinical practice. Since the women’s clinical condition and pregnancy outcome were uneventful, this suggests either that before pregnancy their deficit was over-replaced to some extent or that the dose increment required for pregnancy is less than usually assumed and GH cessation could have contributed. Overall, it seems clear that in women requiring glucocorticoid replacement, a systematic dose increase during pregnancy is not needed. In this line, even when information of the glucocorticoid dose given during pregnancy is usually not reported in articles dealing with adrenal insufficiency during pregnancy [35], in a recent report of 128 pregnancies, glucocorticoid dose was decreased after initial increase in some women and was not modified during pregnancy in a sizeable subgroup of women. In this study only 25% of the participants had secondary insufficiency. The increase in hydrocortisone equivalent during pregnancy was similar in women with Addison’s disease and secondary adrenal insufficiency but this observation might not be fully applicable to our study (i.e. information on concomitant treatment with GH was lacking) [36].

During follow-up, we detected 4 new hormonal deficits that were not present at the beginning of pregnancy and 3 of them resolved after delivery: One patient-initiated treatment with hydrocortisone, one patient required thyroid substitution at week 32 as the free T4 levels were below the normal range, and 2 patients required vasopressin substitution as they developed diabetes insipidus (DI). The patient with a diuresis >5000 mL/24 h, continued requiring desmopressin treatment after delivery, suggesting that diabetes insipidus was already present before pregnancy. Regarding vasopressin substitution, all the patients with ADH deficit diagnosed before or during pregnancy, needed to increase the desmopressin dosage during follow up. In women already treated before pregnancy, the dose increment at the end of pregnancy was 40% (60 mcg). It is remarkable that one of the patients initiating treatment during pregnancy, required up to 360 mcg/day at the time of delivery. It is well-known that pregnancy may unmask a partial DI not clinically relevant before gestation. Several possibilities might explain why pregnancy itself may cause exacerbation of DI. On the one hand, the higher levels of progesterone and steroids antagonize ADH [37]. On the other hand, placental vasopressinase leads to accelerated metabolism of endogenous ADH and vasopressin requirements increase [38]. Additionally, compression of the posterior pituitary by the enlarged anterior pituitary has also been described, aggravating situations of partial DI [39].

Oxytocin is a neuropeptide synthetized in the hypothalamus and released to the neurohypophysis, that stimulates uterine smooth muscle contraction during labor. In recent years several case series have reported spontaneous labor initiation in pregnant women with HYPO and ADH deficiency [40, 41], without requiring oxytocin administration for labor initiation or augmentation in some women, suggesting that endogenous pituitary oxytocin may not be mandatory for spontaneous labor initiation or that it was (partially) preserved. Our results differ from previously mentioned data. Four patients had spontaneous initiation of labor but none of them had ADH deficiency. In the 4 patients with ADH deficiency delivery took place either through elective cesarean section or through vaginal delivery that required labor induction as well as oxytocin administration for augmentation, pointing to a likely deficit of endogenous and necessary oxytocin. Overall, the rate of elective delivery was 67%, nearly double that reported for Spain for the period 20101–2018 [42] but close to the 57% in the center for high-risk pregnancies. Reasons were varied but in one third of cases, it was prompted by the endocrinological condition of the mother.

There is a general consensus on the association of HYPO with maternal-fetal complications such as high rate of miscarriage, pregnancy-induced hypertension, placental abruption, premature birth, and postpartum hemorrhage [18]. However, in this study we did not observe major obstetrical or fetal complications.

We observed no miscarriage in the study group. It is certainly possible that this occurred if some pregnancies went unnoticed to the woman or were not reported to the health care provider. But considering that most patients were under follow-up in the department since before pregnancy, we consider this was likely not a common situation.

Weight gain was excessive in 9 women and adequate in 3. In particular, the patient who gained the most weight (#7) during pregnancy (up to 27 kg), was not on hydrocortisone replacement since she did not have adrenal insufficiency. On the other hand, the second patient (#1) who gained more weight during pregnancy (19.3 kg) presented with panhypopituitarism and hydrocortisone doses remained unchanged throughout all pregnancy. Certainly, inadequate hormonal replacement therapy (cortisol, T4, GH) could potentially have an influence on excessive weight gain; however, in our series, hydrocortisone dose was not high and its increment during pregnancy was negligible, so it is unlikely to be related to excessive weight gain. Additionally, stopping GH therapy at pregnancy confirmation might have a role in weight gain as discussed above. To our knowledge, our study reports and evaluates maternal weight during pregnancy in women with HYPO according to Institute of Medicine guidelines for the first time. This data emphasizes the importance of close maternal weight monitoring during pregnancy, as well as more studies addressing this outcome and related variables such as hormone substitution.

Moreover, all but one delivery was at term and birth weight centiles displayed a normal distribution. Our observation is similar to other studies showing normal weight in the majority of the newborns [6, 12, 14] notwithstanding, other studies in hypopituitary women reported up to half of the newborns small for gestational age [17, 18]. In this regard, it is relevant that in their systematic review, Vila et al. mention that most reports did not provide details on birthweight and gestational age [6]. At delivery, no intrapartum fetal distress was observed, and the median arterial/venous cord blood pH were within the normal range. Nearly half of the reported arterial cord pH values were lower than 7.20, which is clearly higher than the published rates [43] but none was below the 7.10 or 7.00 problematic cut-offs [44, 45]. Regarding hypertensive disorders of pregnancy, it was diagnosed in only two patients.

Our observations are comparable to those of Correa et al. [14], and Sowithayasul et al. [46] who both described a 60% rate of cesarean deliveries. Nevertheless, we consider that the rate of cesarean section in this series was high, considering that the rate in the background obstetric population in the corresponding period was 24.8%.

At the time of hospital discharge, half of the patients were breastfeeding. This figure compares with other reports in the literature [14, 46] and even when the rate is far from satisfactory and that we do not have data on breastfeeding at longer term, women can be reassured that breastfeeding can be possible. In fact, in the last years, women have actively asked this question during pregnancy follow-up. We do not have information about breastfeeding being exclusive, predominant or mixed. But even if rates of exclusive breastfeeding were low, benefits are probably present [47].

The present study has some limitations such as the limited sample size and the descriptive methodology. Further studies with a larger number of patients and whenever possible, randomized trials are needed in order to determine if the actual practical recommendations for pregnant women with HYPO should be modified.

In conclusion, in this case-series of 12 pregnancies of women with HYPO, no relevant maternal-fetal complications were observed apart from a high rate of excessive gestational weight gain and cesarean section. Hormonal dosage adjustment was performed mainly according to clinical judgment without hormonal measurement, except for the thyroid axis. Our findings reinforce the idea that a strict follow up is needed during pregnancy in hypopituitary women in order to diagnose early any eventual new onset hormonal deficit.

## Data Availability

The datasets generated during and/or analyzed during the current study will be publicly available.
